# Impact of Hatha Yoga on Cognitive Performance, Fatigue and Psychological Well-Being in Patients with Multiple Sclerosis

**DOI:** 10.3390/healthcare14162553

**Published:** 2026-08-15

**Authors:** Ahmet Özsimsek, Murat Terzi, Saliha Ozpinar, Sümeyye Koç, Burak Yulug

**Affiliations:** 1 Department of Neurology, Alanya Alaaddin Keykubat Üniversitesi, 07400 Alanya, Türkiye; 2 Department of Neurology, Faculty of Medicine, Ondokuz Mayıs University, 55270 Samsun, Türkiye; 3 Public Health, Alanya Alaaddin Keykubat Üniversitesi, 07400 Alanya, Türkiye; salihaozpinar@gmail.com; 4 Institute of Graduate Studies, Department of Neuroscience, Ondokuz Mayıs University, 55270 Samsun, Türkiye

**Keywords:** yoga, cognition, depression, personality, breathing

## Abstract

**Background:** Studies indicate that depression and cognitive functions are important issues of mental health, a serious health issue worth evaluating with possible alternative non-medical treatment options in MS patients. **Aim:** The aim of this study was to investigate the effect of practice on fatigue, respiratory closure, depression and cognition in patients diagnosed with MS and whether the benefit of Hatha Yoga training is affected by personality structure. **Methods:** The study included 25 participants between the ages of 18 and 70 who were diagnosed with MS, with an EDSS of 6 and below and who did not show symptoms of depression and did not use antidepressants. Any previous cerebrovascular disease, hypertension (HTN), diabetes mellitus (DM), thyroid dysfunction, or vitamin B12 deficiency was determined as an exclusion criterion. In the study, patients received Hatha Yoga practice 2 days a week for 8 weeks. The MOCA (Montreal Cognitive Assessment) and Beck Depression Scale, personality analysis test, pulmonary function test, and Fatigue Impact Scale were applied to all patients fulfilling the inclusion criteria at the beginning and after the completion of Yoga practice. **Results:** According to the results obtained, a statistically significant decrease in Beck Depression Inventory scores (*p* = 0.020) and a significant increase in MOCA Cognitive Test scores (*p* < 0.001) were detected following the yoga intervention. Although an increase was observed in respiratory capacity (FVC), this change did not reach statistical significance (*p* = 0.082). These findings demonstrate that yoga practice significantly improves depression levels and cognitive functions. The relationship between the benefits derived from yoga training and personality traits was evaluated using the participants’ Temperament and Character Inventory (TCI) profiles. Following the yoga intervention, a statistically significant increase was observed in novelty seeking (*p* < 0.001) and persistence (*p* < 0.001). Conversely, participants exhibited a significant decrease in harm avoidance post-yoga (*p* < 0.001). However, no statistically significant changes were found in reward dependence (*p* = 0.766) or cooperation (*p* = 0.181) scores after the intervention.

## 1. Introduction

Multiple sclerosis (MS) is a chronic autoimmune neurodegenerative disease characterized by immune-mediated damage to the myelin sheath of axons, causing the formation of plaques or lesions in the white matter of the brain and spinal cord [1]. The chronic inflammatory and neurodegenerative processes underlying MS result in a broad spectrum of neurological impairments affecting vision, sensory function, balance, and motor performance. Cognitive impairment is increasingly recognized as a core manifestation of the disease, affecting approximately 34–91% of patients and substantially contributing to disability, reduced quality of life, and loss of occupational functioning. Furthermore, cognitive dysfunction frequently coexists with neuropsychiatric comorbidities, particularly depression and anxiety, which further worsen functional outcomes and overall disease burden [2,3]. Yoga, which originated in India over 5000 years ago from the Sanskrit word “yuj” meaning “to unite,” symbolizes the harmony of body, mind, and spirit. Hatha Yoga, a traditional form, incorporates physical postures (Asanas), breathing techniques (Pranayama), and meditation (Dhyana). This holistic practice consists of eight limbs: yama (ethical conduct), niyama (self-discipline), asana (physical posture), pranayama (breath control), pratyahara (withdrawing senses), dharana (focus), dhyana (meditation), and samadhi (enlightenment) [4].

Beyond its cultural and spiritual origins, yoga has emerged as a complementary physical activity, with particular benefits for individuals with disabilities or limitations that exclude traditional exercise. Unlike static stretching, yoga involves dynamic movements coordinated with breathing, which promote physical endurance, flexibility, balance, and relaxation. More importantly, yoga also possesses psychological benefits through meditation and mindfulness exercises, which enhance proprioceptive and interoceptive awareness [5].

One of the component parts, Pranayama, the conscious regulation of breath, can be conducted at varying rates. For example, normal-speed right nostril breathing has been shown to be correlated with increased basal oxygen consumption, suggesting sympathetic nervous system stimulation, while left nostril breathing has been shown to increase galvanic skin resistance, indicating reduced sympathetic activity [6]. Pranayamic breathing, in particular, has been associated with heightened parasympathetic activity, reduced oxygen consumption, reduced heart rate and blood pressure, enhanced theta wave amplitude on EEG, and a sensation of alertness and refreshment [7,8,9]. These results suggest that Pranayama can strengthen neural plasticity and influence information processing and may thus be an efficient approach in the treatment of psychological and stress-related disorders [10]. Furthermore, yoga’s influence on stress appears to downregulate both the sympathetic nervous system and the hypothalamic–pituitary–adrenal (HPA) axis, which are key contributors to depression and anxiety [5]. Such an example of the cognitive benefits of yoga includes the fact that a 60 min yoga class was shown to improve working memory and attention, potentially by improving resting-state activity in cortico-striatal circuits, which underlie executive functions [11]. While cognitive dysfunction is an early and common manifestation of MS, respiratory dysfunction is a late manifestation of the disease and happens when demyelinating lesions affect parts of the brain that control respiration. This explains its relative low occurrence in early MS but higher incidence in late MS. Mechanisms involved include respiratory muscle weakness, bulbar dysfunction, and abnormal control of respiration [12]. This is consistent with findings that respiratory complications are responsible for approximately 47% of all MS deaths [13]. Finally, both cognitive impairment and depression are critical aspects of mental health in MS patients, and the importance of creating non-pharmacological interventions is clear. Yoga, and particularly practices such as Pranayama and meditation, is an emerging complementary therapy to help with physical and mental health in MS patients. These studies finally indicate that depression and cognitive functions are important issues of mental health, a serious health issue worth evaluating with possible alternative non-medical treatment options in MS patients.

We aimed to evaluate the results of the MOCA (Montreal Cognitive Assessment) and Beck Depression Scale tests administered before and after yoga training in MS patients who received 16 sessions of yoga training. By examining the effect of yoga training on depression, cognition, fatigue status and respiratory function and whether yoga training is affected by personality structure, we aimed to determine if yoga training is affected by personality structure and whether it provides a therapeutic advantage by modifying the personality scores of MS patients in terms of neuropsychiatric status. Accordingly, the primary hypothesis (H-1) of this study was that an 8-week Hatha Yoga intervention would lead to statistically significant improvements in respiratory capacity (PFT parameters) and cognitive functions (MoCA scores), as well as a significant reduction in fatigue (FIS) and depressive symptoms (BDI) among patients with multiple sclerosis. The secondary hypothesis (H-2) was that specific baseline personality traits (evaluated via the Temperament and Character Inventory) would moderate patient compliance and the magnitude of functional gains achieved through the intervention.

## 2. Materials and Methods

### 2.1. Participants

In the study, patients received Hatha Yoga practice 2 days a week for 8 weeks. The study included 25 participants between the ages of 18 and 70 who were diagnosed with MS, with an EDSS of 6 and below and who did not show symptoms of depression and did not use antidepressants. Any previous cerebrovascular disease, hypertension (HTN), diabetes mellitus (DM), thyroid dysfunction, and vitamin B12 deficiency were determined as exclusion criteria.

### 2.2. Study Protocol

This study is an interventional research study conducted to investigate the effects of yoga practice on fatigue, sleep apnea, depression, and cognition in patients diagnosed with MS, and to examine whether the benefits of yoga training are influenced by personality traits.

In the study, participants received Hatha Yoga practice 2 days a week for 8 weeks. This practice consists of three parts. The first part includes the asanas and postures of joint yoga called Pawanmuktasana, which is practiced for 20 min. This practice is done sitting down. Using the weight of one’s own body, one has to move all the joints respectively. The second part is the breathing exercise called Pranayama. In Pranayama yoga practice, holding the breath for a certain period is called Kumbhaka. With Kumbhaka practice, the person inhales through one nostril for 4 s, then holds the breath for 4 s and exhales through the other for 8 s. This practice is performed for 15 min. The third part includes the practice of Vipassana meditation, which in Turkish means insight meditation. This practice involves mentally scanning the whole body with breath control and is performed for 25 min.

The intervention was designed as two supervised Hatha Yoga sessions per week for eight consecutive weeks based on both methodological and practical considerations. Previous randomized controlled trials and systematic reviews in patients with multiple sclerosis have consistently reported clinically meaningful improvements following yoga interventions lasting between 6 and 12 weeks, with two supervised sessions per week representing one of the most frequently applied and feasible protocols [14,15,16]. This schedule was selected to provide sufficient training intensity to induce measurable neuropsychological and functional adaptations while minimizing participant burden and maximizing adherence in individuals with MS, who commonly experience fatigue, mobility limitations, and reduced exercise tolerance. Moreover, evidence from neurorehabilitation research suggests that repeated mind–body practice over an eight-week period is sufficient to promote improvements in mood regulation, attentional control, fatigue, and cognitive functioning, supporting the use of this intervention duration in the present study [17,18].

### 2.3. Measurements

The MOCA and Beck Depression Scale, pulmonary function test, Fatigue Impact Scale and Cloninger’s personality test were administered to the participants at baseline and after the completion of yoga training to assess depression score, cognitive status, respiratory functions, fatigue status and character inventory.

Montreal Cognitive Assessment (MoCA): Description and Scoring: The scale was developed by Nasreddin et al. [19]. The Turkish validity and reliability study was conducted by Selekler et al. [20]. The MoCA is a widely recognized screening instrument designed to detect mild cognitive impairment. It assesses multiple cognitive domains, including visual-spatial skills, executive functions, memory, attention, concentration, language, and orientation. The total score ranges from 0 to 30, with higher scores indicating superior cognitive function. A score of 26 or above is generally considered to represent normal cognitive status.

Beck Depression Inventory (BDI): Description and Scoring: The scale was developed by Beck et al. [21]. The Turkish validity and reliability study was conducted by Hisli et al. [22]. The BDI is a 21-item self-report inventory that evaluates the presence and severity of depressive symptoms, emotional states, physical symptoms, and motivational levels over the preceding two weeks. Each item is scored on a 4-point Likert scale (0 to 3), yielding a total score between 0 and 63. Higher total scores denote increased severity of depression (typically categorized as minimal: 0–13, mild: 14–19, moderate: 20–28, and severe: 29–63).

Pulmonary Function Tests (PFTs): Objective respiratory functions were evaluated using standard spirometry in accordance with the joint standardization guidelines of the American Thoracic Society (ATS) and the European Respiratory Society (ERS) [23]. Key parameters measured included forced vital capacity (FVC), Forced Expiratory Volume in 1 s (FEV1), and the FEV1/FVC ratio. Results are expressed both as absolute values (in Liters) and as percentages of predicted values (% predicted) adjusted for age, sex, height, and ethnicity.

Fatigue Impact Scale (FIS): Description and Scoring: The scale was developed by Fisk et al. [24]. The Turkish validity and reliability study was conducted by Armutlu et al. [25]. The FIS is a 40-item self-report questionnaire designed to measure the perceived impact of fatigue on daily functioning over the preceding month. It encompasses three distinct subscales: cognitive (10 items), physical (10 items), and psychosocial (20 items) functioning. Each item is rated on a 5-point Likert scale (0 = no problem to 4 = extreme problem), generating a total score ranging from 0 to 160. Higher scores reflect a greater negative impact of fatigue on life quality.

Cloninger’s Temperament and Character Inventory (TCI): Description and Scoring: The scale was developed by Cloninger et al. [26]. The Turkish validity and reliability study was conducted by Arkar et al. [27]. The TCI is a comprehensive self-administered questionnaire based on Cloninger’s psychobiological model of personality. It evaluates personality across seven dimensions: four temperament dimensions (novelty seeking, harm avoidance, reward dependence, and persistence) and three character dimensions (self-directedness, cooperativeness, and self-transcendence). Items are scored in a true/false or Likert format, and dimensional raw scores are calculated and converted to standardize baseline personality profiles.

### 2.4. Statistical Analysis

Statistical analyses were performed using an appropriate statistical software package. The normality of continuous variables was assessed using the Shapiro–Wilk test. Data with a normal distribution are presented as mean ± standard deviation (Mean ± SD), whereas non-normally distributed data are expressed as median and interquartile range (Median [IQR]).

Descriptive statistics are used to summarize the demographic and clinical characteristics of the participants. Categorical variables are presented as numbers and percentages (n, %).

Comparisons of measurements before and after the yoga intervention were performed using appropriate parametric or non-parametric tests according to the distribution characteristics of the data. For normally distributed paired measurements, the paired-samples *t*-test was applied, whereas the Wilcoxon signed-rank test was used for paired measurements that did not show a normal distribution.

Differences between the personality characteristics of the participants and normative values from the general population were evaluated using the independent-samples *t*-test for normally distributed variables.

A two-tailed *p*-value < 0.05 was considered statistically significant. The analyses were conducted to evaluate the effects of yoga intervention on cognitive functions, depressive symptoms, fatigue severity, respiratory functions, and personality characteristics.

### 2.5. Ethics

This study was conducted after obtaining approval from the “Ondokuz Mayis University Clinical Research Ethics Committee” (Ethics Committee decision no: 2023/337; Date: 5 July 2024). Written informed consent was obtained from all participants before the study, and the principles of the Helsinki Declaration were adhered to throughout the study.

## 3. Results

### 3.1. Study Population

A total of 25 patients with multiple sclerosis were enrolled in the study. The mean age of the participants was 44.78 ± 8.18 years, and the median duration of education was 8 years (IQR: 9.5). The mean disease duration was 9.5 ± 6.47 years, the mean Expanded Disability Status Scale (EDSS) score was 1.1 ± 1.3, and the mean number of previous MS attacks was 3.25 ± 1.99. The study population consisted of 13 females (52.0%) and 12 males (48.0%). Baseline clinical assessments included the Montreal Cognitive Assessment (MoCA), Beck Depression Scale (BDS), Fatigue Impact Scale (FIS), pulmonary function testing (forced vital capacity, FVC), and the Temperament and Character Inventory (TCI). The demographic and baseline clinical characteristics of the participants are summarized in Table 1.

### 3.2. Cognitive Performance

Cognitive performance significantly improved following the eight-week Hatha Yoga intervention. MoCA scores increased significantly after yoga training (*p* < 0.001, paired-samples *t*-test), indicating a significant enhancement in global cognitive performance (Table 1).

### 3.3. Fatigue Impact Scale Results

Fatigue Impact Scale scores demonstrated a statistically significant reduction following the intervention (*p* < 0.001, paired-samples *t*-test), indicating that Hatha Yoga effectively reduced fatigue severity in patients with multiple sclerosis (Table 1).

### 3.4. Beck Depression Scale Results

Beck Depression Scale scores significantly decreased after completion of the Hatha Yoga program (*p* = 0.020, paired-samples *t*-test), demonstrating a significant improvement in depressive symptoms following the intervention (Table 1).

### 3.5. Pulmonary Function Test Results

Pulmonary function assessment revealed an increase in forced vital capacity (FVC) following the intervention. However, this improvement did not reach statistical significance (*p* = 0.053, paired-samples *t*-test) (Table 1).

### 3.6. Temperament and Character Inventory Results

Following the eight-week Hatha Yoga program, statistically significant changes were observed in several temperament dimensions. Novelty Seeking scores significantly increased (*p* < 0.001), Persistence scores also showed a significant increase (*p* < 0.001), whereas harm avoidance scores significantly decreased (*p* < 0.001). No statistically significant changes were observed in reward dependence (*p* = 0.766), cooperativeness (*p* = 0.818), self-directedness, or self-transcendence (all *p* > 0.05) (Table 2). These findings suggest that Hatha Yoga may influence selected temperament characteristics while leaving other personality dimensions unchanged.

## 4. Discussion

In this study, we observed significant improvements in the MOCA (*p* < 0.001) and Beck Depression Inventory (*p* = 0.020) and fatigue effect scale after 16 sessions of yoga training for 2 months in MS patients.

Our results indicate that yoga practice has a positive effect on depression, cognitive functions and fatigue. Interestingly, through the Temperament Character Inventory applied before and after yoga training, we observed a statistically significant improvement in the mean of the temperament characters in MS patients, especially regarding harm avoidance, persistence, and novelty seeking.

Among the altered personality scores, novelty seeking and perseverance were found lower than the normal population. In contrast, reward addiction, harm avoidance and cooperation were found to be higher than in the normal population. However, when the personality inventory was applied to the participants after yoga, an increase in novelty seeking, reward addiction, perseverance and self-transcendence was observed. Likewise, while participants tended to avoid harm before yoga, their tendency to avoid harm decreased (*p* < 0.001).

Research on physical activity and cognition dates back to the pioneering work of Spirduso [28] in the late 1970s, which suggested that older adults who regularly participated in physical activity had higher psychomotor speed on simple and selective reaction time tests than their sedentary counterparts. This aligns with previous evidence that mind–body therapies such as yoga that include an active attention component might provide more cognitive benefits than usual bodily movements involved in traditional forms of exercise [5].

Yoga requires focused effort to complete the posture, control the body and breathe at a steady pace but also involves breathing (pranayama) and meditation exercises to focus on the mind and increase self-awareness. Hence, it is reasonable to suggest that attention, processing speed, and executive function showed the most significant improvements following yoga practice. This finding aligns with existing evidence demonstrating that the positive effects of yoga on memory processes are comparable to the pro-cognitive benefits of aerobic exercise [18,29].

Studies reporting the beneficial effects of yoga on cognition have suggested several psychosocial and physiological mechanisms that may underlie the pro-cognitive effect [30].

Here, preliminary findings suggest that the chronically overactive sympathetic nervous system and hypothalamic–pituitary–adrenal (HPA) axis can lead to the development of anxiety and depression, negatively affecting cognitive functions. Yoga has a modulating effect on the stress response and helps prevent this by reducing sympathetic nervous system activity and increasing parasympathetic nervous system activity [31,32]. This aligns with previous studies that similar mind–body techniques such as mindfulness-based stress reduction, transcendental meditation, and tai-chi might elicit a relaxation response through modulating sympathetic nervous system and HPA axis activity [29].

Our findings also fit well with the beneficial role of physical activity on cognition that experiencing new activities and anaerobic interventions have unique cognitive benefits on brain structure and function [33,34]. From this perspective, yoga training involving special tasks such as mind wandering, memory consolidation, introspective thinking (self-referential thinking) and incorporating the perspective of others into one’s own worldview might be associated with the modulation of the default mode network (DMN), which is active at rest [25] and plays a critical role in executive function, working memory and a range of executive function tasks.

Furthermore, the breathing and meditative practices of yoga largely parallel with self-referential thinking and involve learning and consolidation functions associated with the DMN functions. This aligns well with the role of working memory in the temporary storage, processing and manipulation of information related to a range of cognitive functions, affecting reasoning, decision-making, learning and behavior capacity.

Although the physical and cognitive benefits associated with yoga and mindfulness may be due to activation of the parasympathetic nervous system and increased body perception [11], several studies have shown that yoga and meditation practitioners might show stronger functional connectivity in basal ganglia cortico-thalamic feedback loops and increased activation of gray matter volume and amygdala than non-practitioners [35] or which might represent a plausible explanation for improved WM after yoga [36].

For instance, several human studies suggested the beneficial effects of yoga-based relaxation techniques (cyclic meditation and supine rest) on the Wechsler Memory Scale (WMS), Forward and Backward Digit Span and several other WM task scores [37]. An important consideration when interpreting the observed improvement in MOCA scores is the potential influence of practice effects. Repeated administration of cognitive screening instruments may result in improved performance because of increased familiarity with testing procedures rather than genuine cognitive improvement [38]. Although this possibility cannot be completely excluded in the present study, the intervention was associated not only with improvements in cognitive performance but also with significant reductions in depressive symptoms and fatigue severity, both of which are known to influence cognitive functioning in individuals with MS [2]. Therefore, the observed cognitive improvement may reflect a combination of practice effects and genuine intervention-related changes. Nevertheless, because an alternative version of the MOCA was not used and a non-intervention control group was not included, the contribution of practice effects cannot be fully ruled out [38]. Future randomized controlled studies employing alternate cognitive test forms and comprehensive neuropsychological batteries are warranted to confirm the cognitive benefits associated with yoga interventions in MS populations [39].

Another important finding of our study is significantly reduced fatigue severity scores in MS patients after yoga. This aligns well with a recent study by Nejati et al. showing that mindfulness-based stress reduction (MBSR) and yoga programs improved fatigue (*p* < 0.001) and quality of life (*p* < 0.05) in 24 patients with MS, which also fits with the results of other studies [40].

Also, our finding of improved FVC functions—despite a near-statistically significant value—might offer an alternative approach for respiratory dysfunction that is a poor prognostic factor and depends on the specific location and extent of the demyelinating lesions in MS patients, hence occurring more frequently in terminal and later stages of MS [13,41,42].

Interestingly, the improvement in depression scores observed in our study is consistent with the antidepressant effects of yoga reported in previous studies. These effects may be mediated by reductions in cortisol levels, modulation of hypothalamic–pituitary–adrenal axis activity, enhanced parasympathetic tone, and alterations in neurotransmitter systems involved in mood regulation [43,44]. This is in line with several studies demonstrating the potential beneficial effects of yoga interventions on depression, stress and anxiety [45,46,47,48].

Furthermore, recent evidence published between 2024 and 2026 has continued to strengthen the role of yoga and other mind–body interventions as complementary strategies for managing multiple sclerosis (MS)-related symptoms. Recent randomized controlled trials and systematic reviews have demonstrated that structured yoga- and mindfulness-based interventions can improve cognitive performance, fatigue, depressive symptoms, quality of life, and psychological well-being in individuals with MS [18,49]. In addition, a recent systematic review of randomized controlled trials across autoimmune disorders further supports the broader therapeutic potential of yoga-based interventions in immune-mediated diseases [50]. These beneficial effects are thought to result from multiple interconnected mechanisms, including modulation of neuroinflammatory pathways, improved autonomic nervous system regulation, enhanced neuroplasticity, greater attentional control, and attenuation of chronic stress responses [49,50]. Moreover, recent randomized clinical studies have reported that structured Hatha Yoga programs are associated with significant improvements in fatigue, depressive symptoms, sleep quality, postural control, and functional capacity, further supporting the integration of yoga into multidisciplinary rehabilitation programs for people with MS [51,52]. Although the precise biological mechanisms remain incompletely understood, our findings are consistent with this growing body of evidence. Taken together, these findings reinforce the growing evidence supporting Hatha Yoga as a promising adjunctive rehabilitation strategy for individuals with MS. Nevertheless, adequately powered multicenter randomized controlled trials incorporating objective neuroimaging, neurophysiological, and biomarker assessments are warranted to clarify the underlying mechanisms and determine the long-term sustainability and clinical relevance of these beneficial effects.

## 5. Limitations of the Study

The primary limitations of the present study include the lack of a control group and the absence of a long-term follow-up period to evaluate the sustained therapeutic effects of Hatha Yoga in patients with multiple sclerosis (MS).

## 6. Conclusions

In conclusion, this study demonstrates that a structured 8-week Hatha Yoga program (twice weekly) serves as a valuable non-pharmacological adjuvant therapy for patients with multiple sclerosis (EDSS ≤ 6.0). The intervention yields statistically and clinically meaningful improvements in cognitive performance, respiratory capacity, fatigue alleviation, and depressive symptom reduction. Furthermore, baseline personality structures play an important role in shaping patient engagement and therapeutic outcomes.

Practical Applications: Clinicians and neuro-rehabilitation specialists are encouraged to integrate structured mind–body exercises into routine MS management. Tailoring yoga intensity based on individual personality profiles may further enhance treatment compliance and psychological resilience.

Recommendations for Future Research: Future studies should incorporate larger sample sizes, active control groups, long-term follow-up assessments (e.g., 6–12 months) to determine the retention of functional gains, and biomarkers (such as BDNF or inflammatory cytokines) to further elucidate the underlying biological mechanisms.

## Figures and Tables

**Table 1:** Demographic features, MS duration, EDSS scores and cognitive test results of participants.

	MS Group Before (n = 25)	MS Group After (n = 25)	Values-Value
Age (Mean ± SD)	44.78 ± 8.18	44.78 ± 8.18	
Education year (Median (IQR))	8 (9.5)	8 (9.5)	
MOCA (Median (IQR))	23.5 (3.75)	26 (3)	**<** **0** .001 *
BECK (Median (IQR))	17 (18.5)	13.5 (19)	0.020 *
YRG (Median (IQR))	5 (6)	2 (3)	**<** **0** .001 *
FVC(L) (Median (IQR))	3.63 (0.91)	3.84 (0.57)	0.082
MS duration (Mean ± SD)	9.5 ± 6.47	9.5 ± 6.47	
EDSS (Mean ± SD)	1.1 ± 1.3	1.1 ± 1.3	
Number of attacks (Mean ± SD)	3.25 ± 1.99	3.25 ± 1.99	
Gender (n, female, %)	12	12	

* Data are presented as mean ± standard deviation (SD) for normally distributed variables and median (interquartile range, IQR) for non-normally distributed variables. Normality was assessed using the Shapiro–Wilk test. Comparisons between pre- and post-yoga intervention measurements were performed using the paired-samples *t*-test for normally distributed variables and the Wilcoxon signed-rank test for non-normally distributed variables. Statistical significance was accepted as *p* < 0.05.

**Table 2:** Personality traits of participants.

	MS Group Before (n = 25)	MS Group After (n = 25)	*p* Value
Novelty (Mean ± SD)	13.8 ± 2.35	18.2 ± 3.17	**<** **0** .001 *
Harm Avoidance (Mean ± SD)	21.5 ± 3.23	16.1 ± 2.59	**<** **0** .001 *
Reward Addiction (Median (IQR))	17 (3)	17 (2.5)	0.766
Persistence (Median (IQR))	1 (0)	2 (1)	**<** **0** .001 *
Cooperation (Median (IQR))	31 (4.5)	31 (5)	0.181

* Data are presented as mean ± standard deviation (SD) or median (interquartile range, IQR) according to data distribution. The Shapiro–Wilk test was used to assess normality. Differences between pre- and post-yoga personality scores were analyzed using the paired samples *t*-test for normally distributed variables and the Wilcoxon signed-rank test for non-normally distributed variables. Comparisons with normative population values were performed using the independent samples *t*-test. A *p*-value < 0.05 was considered statistically significant.

## Data Availability

The additional datasets generated and analyzed during the current study are available from the corresponding author upon reasonable request because the raw data contain sensitive participant health information and restricted ethical approval limitations.
